# A Visual Analogue Scale Exercise for Teaching Antimicrobial Selection in Veterinary Pharmacology: Tool Development and Initial Implementation

**DOI:** 10.1111/jvp.70088

**Published:** 2026-06-17

**Authors:** Carly A. Patterson, Jordan Tayce, Johanna C. Heseltine

**Affiliations:** ^1^ Department of Veterinary Physiology and Pharmacology, College of Veterinary Medicine and Biomedical Sciences Texas A&M University College Station Texas USA; ^2^ Department of Veterinary Integrative Biosciences, College of Veterinary Medicine and Biomedical Sciences Texas A&M University College Station Texas USA; ^3^ Department of Veterinary Small Animal Clinical Sciences, College of Veterinary Medicine and Biomedical Sciences Texas A&M University College Station Texas USA

**Keywords:** antimicrobials, clinical reasoning, education, pharmacology, veterinary

## Abstract

Veterinary students often find it difficult to select antimicrobial drugs for patients, likely because it requires them to consider multiple factors and there are frequently several possible options with a lack of a defined “correct or incorrect” choice. Our goal was to develop a teaching tool to engage the students through the decision‐making processes associated with antimicrobial drug selection in cats and dogs, designed to align with the CBVE competency of explaining a justification. We developed a series of small animal case vignettes with a set of antimicrobial choices. The students use a visual analogue scale (VAS) to indicate the relative safety and/or efficacy of the drug in question; they also provide a written justification for their selection. Student responses are anonymized and downloaded for instructor review. The instructor categorizes the frequency of selections according to the labeled quartiles and displays the results as a bar graph during the classroom session and selects some written justifications for the class to view as a group. Practical considerations for tool implementation include considerations of the curriculum, time spent reviewing individual answers, and the tool's utility as a supplementary aid to foster more student discourse about topics in pharmacology. Overall, the VAS tool has the potential to aid in generating discussion in clinical contexts where there is not a single best answer.

## 1 Background and Rationale

Clinical reasoning is a complex skill that requires students to integrate their own knowledge with patient information to make decisions impacting patient care (Gruppen 2017). Therapeutic reasoning is a subset of clinical reasoning that focuses on treatment selection (Abdoler et al. 2020). Teaching therapeutic reasoning related to antimicrobial selection has important consequences in veterinary medicine because of the implications for patient care and responsible antimicrobial stewardship. Since clinical reasoning can encompass many facets and requires practice by the learner, active learning strategies offer potential benefits, such as increased learner engagement with the material and improved overall knowledge base (Tayce et al. 2021). It can be difficult for early learners to select appropriate empirical antimicrobial drugs. Selection of appropriate antimicrobials requires the learner to consider multiple factors including spectrum of coverage (which should be as narrow as possible in alignment with antimicrobial stewardship principles); efficacy at the site of infection; route of administration; potential adverse effects; and sometimes cost (Caneschi et al. 2023). Applying these principles for multiple antimicrobials and clinical case scenarios requires complex decision‐making, and other teaching techniques, such as a metacognitive mnemonic framework and educational games, have been described to try to address the complexities (Cole et al. 2021; Buur et al. 2013).

The American Association of Veterinary Medical Colleges (AAVMC) Competency‐Based Veterinary Education (CBVE), version 2.0, targets learning outcomes that include adapting a treatment plan based on clinical information despite uncertainty (AAVMC Council on Outcomes‐based Veterinary Education et al. 2024). An additional competency of the AAVMC CBVE is that the learner be able to explain the justification of the plan. Our goal was to develop a teaching tool to support the students through the decision‐making processes associated with antimicrobial drug selection in cats and dogs, supporting the CBVE competency of explaining a justification. The teaching tool was designed to provide students with an opportunity to practice applying clinical reasoning to antimicrobial selection while they are concurrently learning about drugs' mechanism of action and adverse effects in the curriculum. This teaching tool uses clinical case scenarios to potentially increase student interest and extrinsic motivation.

The teaching tool is aimed at helping students move beyond fact recall by giving them the opportunity to apply their knowledge to clinical decision making. The case‐based format is designed to provide students with an understanding of the relevance of the content material, because the necessary thought processes parallel those they will use as veterinary professionals for real‐life cases (Frèrejean et al. 2025). Students may experience a high cognitive load because the treatment options are complex and also integrate uncertainty, as available options often have advantages and disadvantages, but there is rarely a single correct answer (Paas and van Merriënboer 2020). To help offset the cognitive load, implementation of the tool is paired with just‐in‐time instruction, fostering an interactive and collaborative discussion between the instructor and the students (Frèrejean et al. 2025). Feedback and guidance can be provided by the instructor about areas they identify as challenging for the learners. The tool provides formative assessment for both the students and the instructor in order to identify areas of weakness that require more clarification or practice.

## 2 Description of the Educational Innovation

Two board‐certified small animal internists (CAP, JCH) considered scenarios that presumably represent cases frequently seen in small animal general practice, for which empirical, systemic antimicrobial selection would be likely and used these to create 9 vignettes (Table 1). A variety of patient signalments and types of infections involving different body systems were included. The vignettes include patient signalment, an abbreviated history, relevant physical examination findings, and the presumptive diagnosis. Each vignette was accompanied by a list of 5 to 7 antimicrobials for the students to evaluate. Some options represented antimicrobials that would be appropriate to use, and others were suboptimal because of factors such as an unnecessarily broad spectrum of coverage, limited efficacy due to pharmacokinetics, or an increased risk of an adverse event in the given patient. For the antimicrobials, route of administration and dosage were not specified, as the authors believed integration of these factors exceeded the students' stage of decision‐making. The aim was to prompt the students to consider factors such as likely causative bacteria, efficacy of the drug at the site of infection as determined by the antimicrobial's pharmacokinetics, breed‐ or species‐related adverse effects, and, in some scenarios, drug interactions.

**TABLE 1:** Signalment, diagnoses, and pre‐determined antimicrobial options provided.

Species and presumptive diagnosis	Antimicrobial options
10‐year‐old MN DSH cat with sepsis	Clindamycin, enrofloxacin, ampicillin, meropenem, chloramphenicol
11‐year‐old FS Chihuahua dog with diskospondylitis	Cephalexin, pradofloxacin, amikacin, chloramphenicol, amoxicillin‐clavulanate
3‐year‐old FS Yorkshire Terrier dog with pyelonephritis	Cefpodoxime, enrofloxacin, gentamicin, trimethoprim‐sulfamethoxazole, amoxicillin‐clavulanate
9‐year‐old MN Dachshund dog with tooth root abscess	Doxycycline, enrofloxacin, clindamycin, cefovecin, amikacin
3‐year‐old FS Labrador retriever dog with bacterial cystitis	Chloramphenicol, trimethoprim‐sulfamethoxazole, enrofloxacin, ampicillin, amikacin
6‐year‐old MN Doberman pinscher dog with bacterial cystitis	Ampicillin, amoxicillin‐clavulanate, enrofloxacin, chloramphenicol, trimethoprim‐sulfamethoxazole
5‐year‐old MN mixed breed dog with superficial pyoderma	Ampicillin, amoxicillin‐clavulanate, enrofloxacin, clindamycin, gentamicin, cephalexin
4‐year‐old FS Dachshund dog with aspiration pneumonia	Trimethoprim‐sulfamethoxazole, enrofloxacin, amoxicillin‐clavulanate, clindamycin, chloramphenicol, amikacin
7‐year‐old MN DSH cat with bacterial cholangitis	Amoxicillin‐clavulanate, imipenem, cefovecin, pradofloxacin, amikacin

Abbreviations: DSH, domestic shorthair; FS, female spayed; MN, male neutered.

A visual analogue scale (VAS) is a measurement instrument that tries to measure a viewpoint that is believed to range over a continuum of values that cannot be objectively measured (Hayes 1921). The VAS tool has been investigated by researchers in various fields, and it is adaptable to different disciplines (Yeung and Wong 2019). The goal was to develop a VAS as a clinical reasoning tool to allow learners to demonstrate a strength of preference for the selected antimicrobials for the given case scenarios. Additionally, each student provides a written justification for their decisions. The aim was to illustrate to students that complex decision making requires integration of factual information and also involves uncertainty. Additionally, the instructor can gather real‐time data about the students' responses and evaluate these to provide collective, formative feedback regarding student decision‐making approaches to antimicrobial drugs.

For each antimicrobial, the learner indicates on the VAS how likely they are to select that antimicrobial for the case outlined in the vignette (Figure 1). The diagram associated with the VAS includes a color gradient from red to yellow to green, paired with a curvilinear diagram increasing from left to right (representing an increasing likelihood that the participant would select the antibiotic). The bar along the x‐axis included labels at the quartiles that read: “not indicated (not safe or not effective)”; “may be used with extreme caution”; “may be used with minor concerns for safety”; and “safe and effective for use in the patient”. Participants are then asked to justify their answer with a free text response. Figure 1 illustrates a case example with one antimicrobial drug. The vignettes are entered into a learning management system (LMS; Canvas) that allows the learner to review the vignettes in series. Students receive credit as a complete/incomplete grade.

**FIGURE 1:**
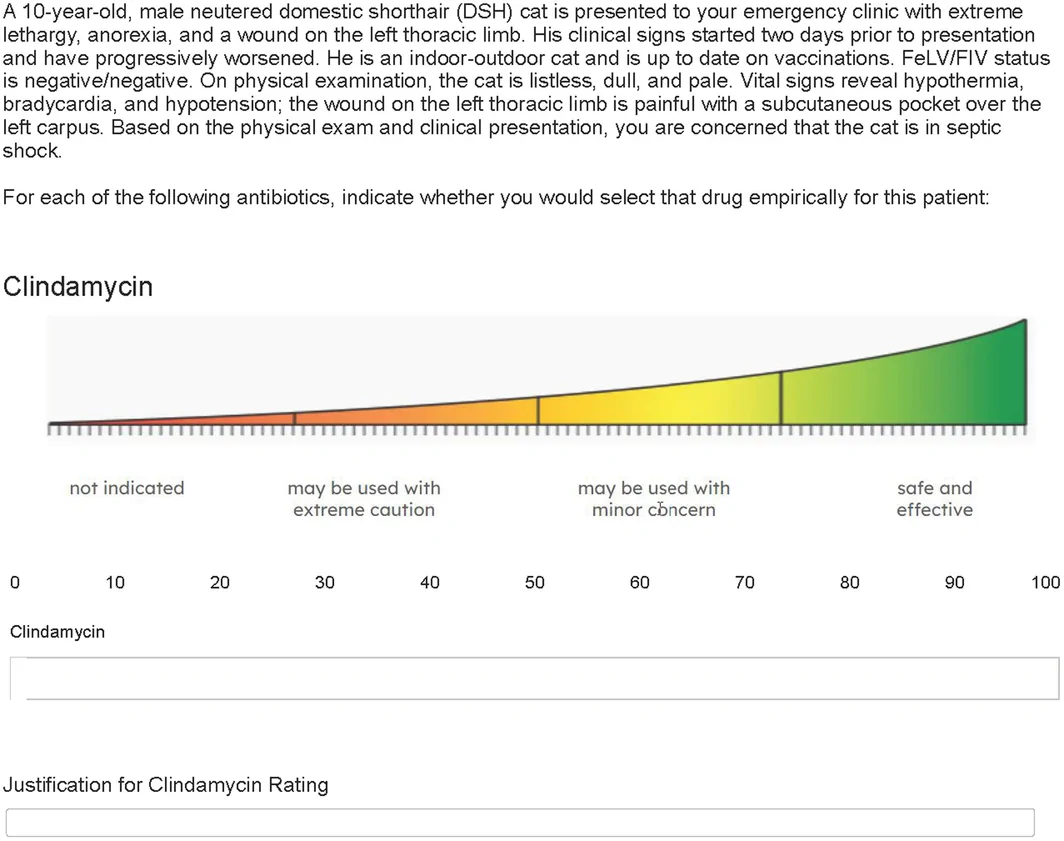
VAS for the septic cat case.

## 3 Implementation Process

The activity is implemented in the fall semester for second‐year students at the Texas A&M College of Veterinary Medicine and Biomedical Sciences (TAMU CVMBS) in two, 2‐h laboratory sessions, each including one half of the class. Because the pharmacology course is team taught and subject to slight variation from year to year, not all of the antimicrobial options are discussed in didactic lectures prior to the VAS activity, but the background principles of antimicrobial stewardship have been covered. The students have 1 week to complete the VAS activity with the free text responses. Students are instructed to work independently and refrain from consulting resources (although use is not explicitly forbidden) when submitting their justifications in order to prevent copied and pasted formulary responses.

Student VAS selections and justifications are anonymized and downloaded into a Microsoft Excel file for instructor review. While the students do not see numerical scores when completing the VAS, the instructor can determine each student's numerical score corresponding to their VAS selection, as well as the student's corresponding justification. The instructor categorizes the frequency of selections according to the labeled quartiles and displays the results as a bar graph during the classroom session.

The instructor selects salient student justifications and presents them anonymously to the class as exemplars of clinical reasoning about antimicrobial selection for the case. Selected responses are those that seem to be original (i.e., not copied verbatim from an online source) and account for the patient signalment, predicted organism(s) at the site of infection, whether therapeutic concentrations are expected at the site, and the potential for adverse effects. The instructor reviews the case vignette with the students and discusses relevant pathophysiology, anticipated pathogens, and potential barriers to treatment. For each antimicrobial, the instructor discusses the mechanism of action and highlights the spectrum of coverage.

## 4 Preliminary Experiences and Observations

The use of data for this publication was granted by the Institutional Review Board at TAMU. To illustrate the teaching activity, an example of the VAS for a septic cat case is shown with one antimicrobial for consideration (Figure 1). All students completed the activity (*n* = 129). The distribution of student responses for all five antimicrobials listed for this case is presented in Figure 2. Selected examples of the free text responses justifying students' choice of antimicrobials are described in Table 2. Individual student's knowledge base and familiarity with the antimicrobials was likely impacted by content that had been covered in the didactic pharmacology course, personal knowledge about the topics, and their prior clinical experiences. The responses were used by the instructor to explain that there is often more than one suitable antimicrobial choice and that the clinician must consider multiple factors when making a selection for a specific patient.

**FIGURE 2:**
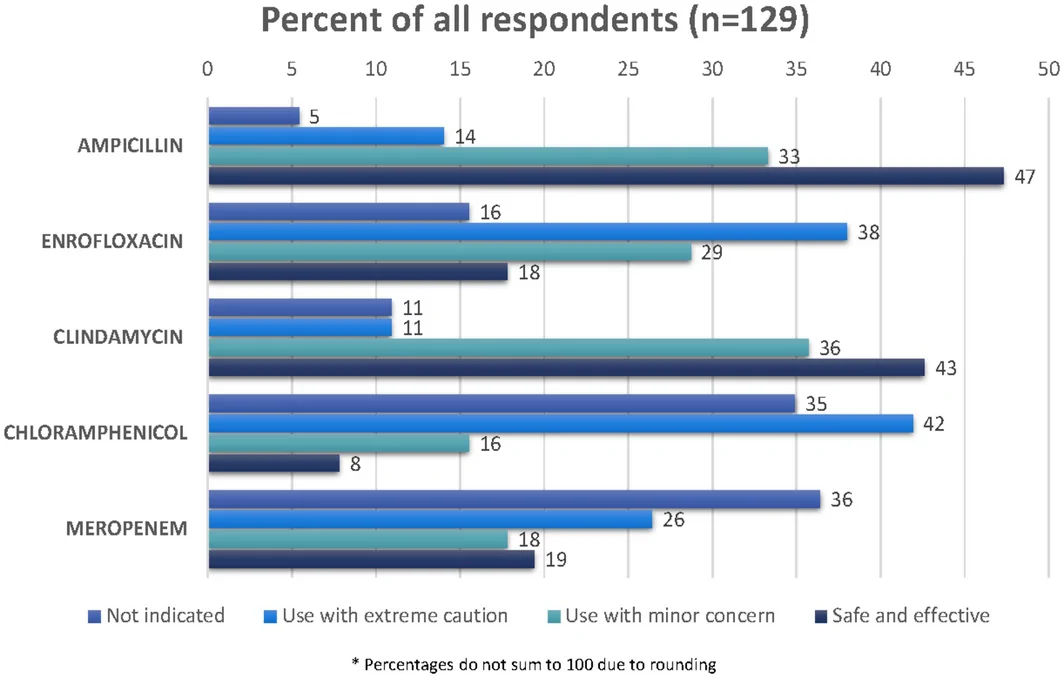
Distribution of student responses by antimicrobial drug for the septic cat vignette.

**TABLE 2:** Selected examples of students' justifications (text in quotations captured verbatim) presented to the class during the septic cat case discussion.

Drug	Justification
Ampicillin	“Ampicillin is an aminopenicillin and effective against Gram positive aerobes and anaerobes and against Gram negative anaerobes and Pasteurellaceae. It is a safe drug, with few reported adverse effects (as long as the animal is not allergic). It is not highly bioavailable when administered orally, but it is not recommended that this drug be given orally in cases of septicemia, so that should not be an issue. I would feel comfortable using this drug empirically.”
Enrofloxacin	“Dangerous if given at a dose greater in 5 mg/kg/day in cats. This can be a good combination with ampicillin for broad spectrum coverage of gram positive and negative bacteria.”
Clindamycin	“The case sounds like an abscess gone septic, which makes me think *Pasteurella multocida* , anaerobes, strep and staph. Clindamycin would cover all except Pasteurellaceae but is not commonly used in cats. Oral forms are associated with esophageal strictures in cats, but since the patient is septic, the IV form would likely be used.”
Chloramphenicol	“Chloramphenicol has broad spectrum action against many aerobic and anaerobic gram + and gram—bacteria. However cats are more sensitive to this drug and can develop adverse effects such as bone marrow hypoplasia because the half‐life of the drug is much longer in cats than it is for dogs. I would not use this drug as my first choice in cats due to the potential for complications.”
Meropenem	“Meopenem although effective against many types of bacteria (gram‐positive, gram‐negative, aerobic, anaerobic, etc.), is never indicated for empirical use and therefore should not be used in this patient.”

Because the VAS allowed students to provide answers along a continuous scale, rather than providing “correct or incorrect” options, it provided the instructor with a large amount of student‐generated data that was easy to organize. Presentation of selected student responses allowed real‐time discussion of the topics that students understood well and of those about which the students demonstrated misconceptions. After the instructor reviewed the VAS results and arbitrarily selected exemplar responses, robust discussion with commentary and questions from the students followed. For example, students discussed their personal observations and experiences from working in various clinical veterinary settings. Additionally, human health implications of veterinary antimicrobial selection were reviewed.

The in‐class discussion was key in ensuring that students completed the activity with resolution of perceived prior misconceptions. As an example, at the end of the laboratory discussion about the septic cat case, meropenem was discussed as an option. Many students described it as an appropriate first‐line choice based on the spectrum of coverage and the cat's critically ill status, despite meropenem being classified as a drug of last resort by the World Health Organization. To further emphasize why this was not an appropriate selection, the instructor provided the students with the final culture and susceptibility report, in this case from a sample collected from the cat's wound. The isolated organism ( *Streptococcus pneumoniae* ) was susceptible to all the antimicrobial drugs on the panel. The instructor used this to reinforce that selection of antimicrobials should be based on likely causative agents and that critical illness is not necessarily the result of a highly resistant organism.

Student feedback regarding the teaching activity was collected as part of end of semester course evaluations deployed to the students. The survey assessed the instructor conducting the session and provided opportunities for students to leave free text comments on the VAS experience. The survey response rate was 24% (35/128) due to attrition of one student during the semester. Overall, the feedback from the students about the use of the VAS in this setting was positive. Some selected examples of student feedback from the septic cat case are provided:

The case assignment really made me think about why things would or would not be good choices and made me work independently!

After completing this activity, I felt so grateful that I had finally been asked to put the concepts of antibiotic selection into action, and make a decision. Then I could receive feedback and learn where my weaknesses still were in choosing a drug. I honestly would really like if we had had this lab sooner in the semester. I went into the first Pharm exam with very low confidence in my ability to apply my antibiotic uses knowledge, and I think it would have been helpful to do an activity like this beforehand.

I really appreciated that you used the comments written by the class for examples of good justification. I think that is just awesome positive reinforcement that we know more then we think we know.

Out of the 35 total survey responses, some students (*n* = 2) expressed frustration that after they submitted their answers, they could not access their responses again for the class discussion. Some students (*n* = 3) wanted to refresh their memory of their answers or thought process during the class session. Modifications to the VAS tool could allow the instructor to capture individual student answers and provide a copy of their responses to each student.

## 5 Practical Considerations

Creation of this teaching tool required the authors to write the clinical vignettes and determine antimicrobial choices to highlight selected key points for the second‐year students. In preparation for the in‐class discussion, the instructor (CAP) needed to review the distribution of the students' responses, as well as evaluate the free text responses in order to identify themes. Because of variations in the students and their prior clinical experiences, timing in the semester, and improved emphasis on challenging topics as the tool was used over sequential years, the topics to highlight vary somewhat from year to year.

The VAS tool was designed in an HTML format by the institutional educational technology support team and integrated with the LMS for ease of student access and the ability to track student completion. The LMS provided inherent ability to download student responses into an Excel sheet for instructor review. Reproduction of the VAS tool by others may vary based on available support staff, resources, and technology.

The tool was designed to help students make decisions about antimicrobial selection despite uncertainty. Interestingly, a few students (*n* = 3) commented directly to the instructor after the laboratory session that they wanted to know the “correct” answer. This emphasized the necessity of continuing to practice antimicrobial decision making with students. Clinical reasoning is a challenging process that requires reinforcement over time, and for some students, a single case exercise using the VAS did not help them grasp the continuum of possible appropriate answers. The authors believe that implementing multiple cases with in‐class discussions over several weeks would further engage student learning.

This tool provided formative feedback for the students and instructor. Responses were anonymized, with the intent of lessening anxiety and stress for the students, which may have helped to offset the high cognitive load associated with complex decision‐making coupled with uncertainty. Both stress and uncertainty can suppress cognitive states of learners (PAAS 2020). Students may have been more comfortable responding because they were not being graded, and their responses were read from an anonymized collective. Because some students could not remember the answers that they provided, they requested to be able to access their responses. Due to limitations accessing student responses within the LMS system after submission, the instructor modified instructions in subsequent years and encouraged students to copy and paste their responses into a separate document prior to submission, which enabled them to refresh their memories during the in‐class discussion session.

## 6 Implementation Guidance for Other Educators

This teaching tool could be readily implemented by other instructors and could be tailored to their area of teaching. For example, cases about food animals could be developed which would add unique considerations regarding appropriate antimicrobial selection. The cases could also be adjusted to align with the students' level of training. For example, because this was used with second‐year students, a case diagnosis was included, but this could be omitted for higher level students, requiring them to make that conclusion based on history, signalment, physical examination, and diagnostic test results. Additional factors that influence decision‐making could also be introduced and added to the discussion synopsis; for example, route of drug administration could be included. If the decision‐making process is too difficult, students may disengage (Frèrejean et al. 2025). If the instructors determine that the decision‐making process is too difficult, the activity could be divided into more steps, having the students indicate the appropriateness of the antibiotic based on individual factors such as pharmacokinetics, efficacy at the site of infection, etc., before asking them to make a global assessment about how likely they would be to select that antibiotic for that patient. An additional possibility is that the tool could be used to teach about patient‐specific selection of drugs other than antimicrobials, such as analgesia selection. Students may not be familiar with use of a VAS or may not have contemplated all of the factors that are incorporated in selection of an antimicrobial, so prior to use of the tool, a demonstration using a case example could allow the instructor to explain the thought process behind their decision‐making.

The just‐in‐time feedback has an important role in this teaching exercise. Use of actual exemplars from students in that specific class may help to make the activity feel authentic and also allow students to self‐assess their decision‐making rationale compared to their peers. To facilitate discussion, the instructor can inquire about prescribing choices that the students have observed in clinical practices, as this allows for assessment of better and worse choices without making the students feel accountable for those choices. This can also emphasize the clinical relevance of therapeutic decision‐making. Because the goal is to generate open dialogue by creating a partnership between the instructor and the students, it helps to remain non‐judgmental when explaining why an antimicrobial is not appropriate or why another is more appropriate. Constructive feedback can help learners understand what they did well and where they can improve, and to make connections between the simulated cases and the real‐life clinical situations they will encounter as professionals.

Students might need to be reminded that case‐based decision making is hard and requires practice, in an effort to normalize their experiences and encourage them to keep trying. The intent is to help prepare the students for the complexity of clinical practice and emphasize the so‐called “shades of grey” inherent in many clinical decisions.

This teaching tool can be readily scaled up (or down) to accommodate for different class sizes. If more students participate, the number of responses the instructor is required to review will also increase. However, compared to the effort invested in developing the vignettes and the teaching tool, the time to compile and review the student responses is relatively small; and once developed, the tool can be used over multiple years.

## 7 Limitations and Future Directions

The described VAS teaching tool has not been validated. This limits any conclusions that can be made about the efficacy of the tool for its intended purpose. The results of the VAS exercise represent student self‐reported learning experiences and are not supported by statistical analysis in this iteration. Additionally, the instructor chose the “salient” examples without a standardized format and there was no systematic thematic analysis. The authors' assessment of the tool and its usefulness is fully subjective without evaluation of learning outcomes. The VAS tool subjectively measured student participation and there is currently no pre‐ or post‐assessment of student competency. Furthermore, the tool has only been implemented at the author's institution and the data were collected from a single class, so the benefits and limitations may vary if it is used at other institutions. The VAS tool was offered to the entire class of students and there was no control or comparison group. There was limited response rate on course evaluation and given the marked overlap in how the subject material is presented in concurrent courses, there is enormous confounding potential. While students were instructed not to use outside resources, overall adherence to the recommendation was not verified. Considering significant limitations, future directions to strengthen the VAS tool include validating it against an expert panel of veterinarians, conducting inter‐rater reliability for scoring responses, and psychometric testing of the VAS scale properties. In the current iteration, the VAS tool is limited to one specific curriculum and LMS platform, which impedes its overall generalizability to other institutions. Future research questions to consider once the VAS tool is properly validated include whether VAS tool performance correlates with exam scores on antimicrobial questions, whether students retain the clinical reasoning skills longitudinally, how the tool itself compares to other active learning modalities, whether it can be successfully implemented at other institutions, and ultimately, does the tool alter attitudes towards antimicrobial stewardship efforts. At this point in the VAS tool development process, it is another potential resource for instructors when discussing cases. It certainly does not supplant other methods for assessing knowledge, such as multiple‐choice questions.

## 8 Conclusions

The authors' goal was to use the VAS as a teaching tool to provide a visual representation of the scope of decision‐making involved in selecting antimicrobials for clinical cases. The aim was to help the students understand that an antimicrobial choice may not be right or wrong but rather requires evaluation of multiple factors that contribute to making a drug more or less appropriate. The goal was to provide students with the opportunity to practice therapeutic reasoning, incorporating the complexities of clinical cases. We intended that the VAS would compel students to actively engage with the material prior to class in a lower stress setting, while the post‐activity discussion would be tailored to address students' strengths and misconceptions that were identified, particularly using their free text responses. The VAS teaching tool has the potential for use in other areas of the veterinary curriculum to generate student discussion and decision‐making about medication selection when there is not a single correct choice.

## Author Contributions

C.A.P., J.T., and J.C.H. contributed to conception, drafting, and review of this article.

## Funding

Funded through an internal grant (Professional student differential tuition, College of Veterinary Medicine and Biomedical Sciences, Texas A&M University).

## Ethics Statement

The authors have nothing to report.

## Conflicts of Interest

The authors declare no conflicts of interest.

## Data Availability

The data that support the findings of this study are available on request from the corresponding author. The data are not publicly available due to privacy or ethical restrictions.
